# Comparing Manual and ChatGPT Deep Research on Systematic Search and Selection in the PubMed Database on the Topic of Dental Implantology

**DOI:** 10.1155/ijod/2677641

**Published:** 2025-10-13

**Authors:** Bulcsú Bencze, Alwin Sokolowski, Jae-Hyun Lee, Péter Hermann, Tamás Hegedüs, Wataru Kozuma, Reo Ikumi, Michael Payer, Ángel-Orión Salgado-Peralvo, Dániel Végh

**Affiliations:** ^1^Department of Prosthodontics, Semmelweis University, Budapest, Hungary; ^2^Department of Dental Medicine and Oral Health, Division of Prosthodontics, Restorative Dentistry and Periodontology, Medical University of Graz, Graz, Austria; ^3^Department of Prosthodontics and Dental Research Institute, Seoul National University School of Dentistry, Seoul, Republic of Korea; ^4^Department of Prosthodontics, School of Dental Medicine, University of Connecticut Health, Farmington, Connecticut 06030, USA; ^5^Department of Oral Implantology and Regenerative Dental Medicine, Tokyo Medical and Dental University, Tokyo 113-8549, Japan; ^6^Division of Oral Surgery and Orthodontics, Department of Dental and Oral Health, Medical University of Graz, Graz, Austria; ^7^Department of Surgery and Medical-Surgical Specialties, Faculty of Medicine and Dentistry, University of Santiago de Compostela, Santiago de Compostela, Spain

**Keywords:** artificial intelligence, ChatGPT, deep research, implantology

## Abstract

**Introduction:**

Dental implantology has seen rapid technological advancements, with artificial intelligence (AI) increasingly integrated into diagnostic, planning, and surgical processes. The release of chat-generative pretrained transformer (ChatGPT) and its subsequent updates, including the deep research function, presents opportunities for AI-assisted systematic reviews. However, its efficacy compared to traditional manual research has not been researched.

**Materials and Methods:**

A systematic review was conducted on May 6, 2025, to evaluate recent innovations in dental implantology and AI. Two parallel searches were performed: one using ChatGPT 4.1's deep research tool in the PubMed database and another manual PubMed search by two independent reviewers. Both searches used identical keywords and Boolean operators targeting studies from 2020 to 2025. Inclusion criteria were peer-reviewed studies related to implant design, osseointegration, guided placement, and other predefined outcomes.

**Results:**

The manual search identified 124 articles, of which 23 met the inclusion criteria. ChatGPT retrieved 114 articles, selected 13 for inclusion, yet only included 11 in its synthesis. Two cited articles by the AI software were nonexistent, and numerous relevant studies were not retrieved, whereas the remaining articles were correct and found by manual search as well. ChatGPT had high specificity (98%) and low sensitivity (47.8%), with a statistically significant difference compared to manual search and selection.

**Discussion:**

AI tools like ChatGPT show promise in literature search, synthesis, and assistance, especially in improving readability and identifying trending topics in science. Nevertheless, the current state of deep research function lacks the reliability required for conducting systematic reviews due to issues such as made-up references and missed articles. The results highlight the need for human supervision and improved safeguards.

**Conclusions:**

ChatGPT's deep research function can support, but not replace manual systematic search and selection. It offers substantial benefits in writing support and preliminary synthesis due to acceptable accuracy, but limitations in reliability and low sensitivity (47.8%) require cautious use and transparent reporting of any AI involvement in scientific research.

## 1. Introduction

Dental implants are important treatment options for the replacement of missing teeth. The success of implant treatment depends on several factors, including implant design, surface characteristics, and implant placement techniques [1]. Over the years, significant advances have been made in dental implantology, resulting in improved clinical outcomes and patient satisfaction. Dental implantology has rapidly evolved over the past few decades, with the introduction of innovative techniques and technologies that have revolutionized the field. Dental implants are now considered the standard of care for replacing missing teeth, and they have shown high success rates and improved long-term stability compared to other options [2]. Recent advancements in artificial intelligence (AI) have significantly influenced dentistry and dental implantology in various aspects, from diagnosis and treatment planning to surgical execution and prosthetic design [3–6].

It only took 2 months after the release of ChatGPT (chat-generative pretrained transformer; OpenAI) to become an extremely popular phenomenon [7]. In response to user input, the chatbot ChatGPT generates realistic text. It is a “large language model” (LLM), a neural network-based system that masters a task by ingesting massive amounts of previously produced human-written text. The technology was made available, partially free, on November 30, 2022 by the software company called OpenAI [8]. ChatGPT is part of the natural language processing (NLP) computer science field, which focuses on the interaction between human and computer language. It can be used for different tasks, such as translating and summarizing texts, creating analyses, and generating images, plots, and figures. ChatGPT has already been used in the scientific field to help authors in the creation of research paper abstracts, summaries, figures, and cover art for research papers. It can also prove to be very useful in the medical field in extracting data from medical records, helping with literature search, and increasing the quality of writing style. The potential of this technology is endless; it could also help in the review and editing process by allowing reviewers to provide real-time feedback on scientific papers [9]. However, this technology has its downsides as well, citing nonexistent literature, generating and spreading misinformation, or prolonging certain stereotypes. Also, the lack of knowledge on the origins of the generated text can present significant challenges. Such sources may raise questions concerning copyright issues, and as such, the technology needs to be given careful consideration to avoid any potential legal implications [10]. ChatGPT received crucial updates that enabled the AI software to analyze data and do deep research and data synthesis. Data analysis was available from August 2023, and recent research investigated its consistency on descriptive statistics and its analytical efficacy, and found great results in comparison with conventional statistical software [11]. A 2025 Cambridge systematic review found that AI tools, compared to human selection, exhibited higher error rates: missing 68% – 96% of studies in searches (median 91%), committing incorrect inclusion/exclusion decisions (median exclusion error 28%), and making 14% – 27% inaccuracies in data extraction [12]. In contrast, a 2024 multicenter study demonstrated effective human–AI collaboration that significantly reduced missing valuable articles in screening to under 1%, achieving accuracy comparable to human reviewers [13]. Deep research is ChatGPT's novel web-research tool. It conducts multistep searches, reads webpages, PDFs (including visuals), and utilizes Python coding and synthesis for analyses and plot generations, ultimately preparing a fully cited report. It is different from normal browsing; hence, it iterates, backtracks, and synthesizes findings with citations. It is built to spend more time on higher-confidence outputs. However, similarly to all models, it has limitations, such as citation misalignments, open-access dependent synthesis, and a lack of protocol integration (e.g., PRISMA).

Deep research became available in February 2025, which enabled the user to conduct systematic searches in certain databases; however, no previous study has investigated the efficacy of deep research compared to conventional manual research.

The primary aim of the current study is to investigate the capabilities of ChatGPT's deep research function in conducting systematic searches for systematic reviews in contrast to a manual systematic search, on the topic of dentistry. The specific choice of the topic, namely, recent advancements in dental implantology and AI, was chosen based on the expertise of the authors to verify the credibility of the generated synthesis, and also the novelty of the topic.

## 2. Materials and Methods

This systematic review was generated with the help of ChatGPT 4.1 Plus on May 6, 2025.

The purpose of this retrospective comparative study was to compare the capabilities of (C) ChatGPT 4.1 deep research plugin on generating a systematic review on the topic of (P) latest innovations in dental implantology and to compare it with (I) human systematic search focusing on specific outcomes such as (O) implant design, implant recognition, surface modifications, immediate loading, computer-guided implant placement, zygomatic implants, future directions, and osseointegration. The topic was chosen based on the expertise of the authors to maintain credibility.

ChatGPT 4.1 deep research function was used to conduct a systematic review, followed by the synthesis of found articles on the topic of “latest innovations in AI and dental implantology.” Prompts previously validated by the authors, based on peer-reviewed suggestions published previously, were used [14]. The language AI was precisely instructed on the task to accurately conduct a systematic search and subsequently conduct a selection on the retrieved articles based on criteria later described. The prompt gave a specific description of the research topic and the PICO framework. It received a validated search key for the systematic search, which included “implant design,” “surface modifications,” “osseointegration,” “immediate loading,” “computer-guided implant placement,” “zygomatic implants,” and “future directions.” Next, ChatGPT was asked to enable deep research and systematically screen the PubMed database for articles published between 2020 and 2025. For further precision, the prompt emphasized the importance of including both open-access and paywalled articles. For the selection procedure, detailed instructions on inclusion and exclusion criteria were given, based on the PICO framework, eligible publication types, and timeframe. All the prompts were given as one input, in order to minimize the variety between different responses and to promote repeatability. The AI search and validation were conducted by one reviewer (B.B.). The generated information made by ChatGPT 4.1 topics was included in this article, written in italics.

A manual search was performed by two authors (T.H. and D.V.) on the same date in the PubMed database using the same keywords that were used as a search key in ChatGPT 4.1, conjoined with the use of Boolean operators. Similarly, only articles from the past 5 years were included.

For both the manual and AI systematic search, similar inclusion and exclusion criteria were defined. The inclusion criteria were peer-reviewed scientific articles, either in vitro studies, observational studies, reviews, or clinical trials published between 2020 and 2025 that included the predefined outcomes. We have excluded articles with inappropriate study types, such as animal studies, abstracts, editorials, preprints, posters, and letters, or if they discussed topics other than the research question.

After conducting the search, duplicate removal, the titles, and abstracts of all identified studies were independently screened by two review authors (T.H. and D.V.). If the articles appeared to meet the inclusion criteria or if there was insufficient data in the title and abstract, the full report was obtained. The selected full texts were then analyzed in duplicate by the same two review authors to make the final decision regarding their inclusion in the study.

Qualitative comparison between the final pools of manual and ChatGPT deep research function was carried out on sensitivity, specificity, and accuracy of AI search and selection assuming manual search and selection as the gold standard. The difference between the manual and deep research pool was tested using McNemar's test. All statistical calculations were carried out with R (version 4.5.1).

## 3. Results

**Figure 1:**
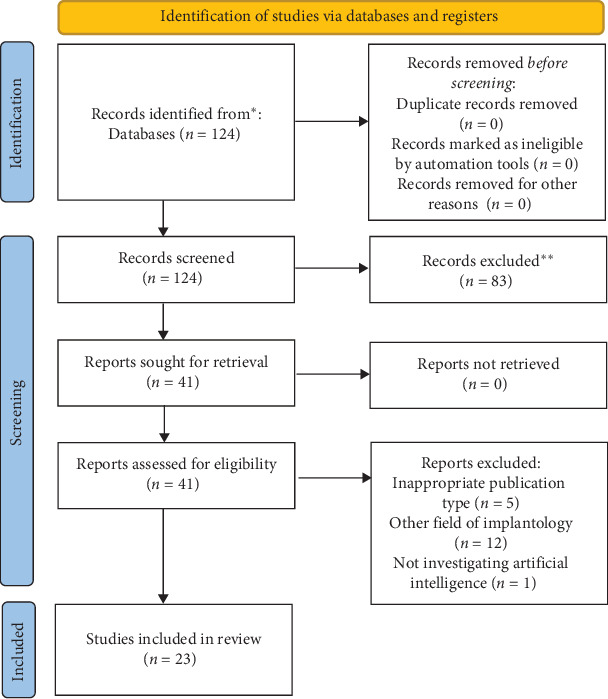
Flowchart of selection (PRISMA 2020). *⁣*^*∗*^The electronic database PubMed was the source for systematic search. *⁣*^*∗∗*^No automatation tools were used for the selection procedure.

The manual search yielded 124 hits from the PubMed database. From that pool, 83 records were excluded after thorough examination of the titles and abstracts of the studies. A total of 41 articles were retrieved for full-text selection, from which 18 were excluded due to inappropriate study design or discussing other fields of dentistry and implantology. We identified 23 eligible up-to-date articles addressing the specific topics of implantology and AI between 2020 and 2025 (Figure 1).

A systematic search on PubMed for studies from 2020 to 2025 using (“dental implant” OR “dental implantology”) AND (“implant design” OR “surface modifications” OR “osseointegration” OR “immediate loading” OR “computer-guided implant placement” OR “zygomatic implants” OR “future directions”) AND (“artificial intelligence”) retrieved 114 records. After removing duplicates and excluding animal studies, conference abstracts, letters, and preprints, 13 full-text articles were eligible for inclusion.

The AI software has listed 11 articles for inclusion; however, it has written that it found 13 articles. From the 13 articles, only 11 were used by the AI software for the synthesis, as it made a mistake of stating that 13 articles existed; however, it did not admit it. It is important to emphasize that the remaining two articles are not real and were made up by the AI software with certain reasons to account for its two missing articles (Table 1).

### 3.1. Implant Design

AI has been applied to optimize implant geometry and materials. Revilla-León et al. [15] found that AI-driven optimization (often combined with finite-element analysis) can significantly reduce stress at the implant–bone interface (up to ~ 36.6%) and fine-tune porosity, length, and diameter. These studies indicate that machine learning and genetic algorithms can help design patient-specific implants that potentially improve stability and longevity. Overall, AI-based design models have shown great potential but remain under development, requiring further clinical validation [15].

One article was missing from the AI conducted systematic search on the topic [16].

The article by Choudhury et al. [16] presents an innovative approach to dental implant design by integrating finite element analysis (FEA) with artificial neural networks (ANNs) to design patient-specific basal dental implants, which assesses stress distribution and mechanical performance of implants under various loading conditions. This hybrid approach allows for rapid evaluation of design parameters without the need for extensive physical prototyping, aiming to enhance the fit and longevity of basal implants [16].

### 3.2. Implant Recognition

AI excels at identifying implant components and diagnosing peri-implant conditions from radiographic images. Recent systematic reviews report high accuracy (>90%) for AI in implant detection and classification on 2D X-rays [17]. Convolutional neural networks have been trained to recognize implant types, detect screw loosening, and even distinguish marginal bone loss or peri-implantitis. For instance, one review found that AI models classified dental implant fixtures with mean accuracies above 90% [17]. In summary, AI-based recognition is a powerful diagnostic aid, enabling automated identification of implant features that support clinical decision-making.

Seven articles were missing from the AI conducted systematic search in the topic [18–24].

A systematic review was conducted on the topic of AI that recognizes implant types, predicts their success, and optimizes implant design [18]. The AI software could identify dental implants from standard periapical and panoramic X-rays with an accuracy of 93.8% – 98% [18]. These AI models could also be used to improve implant designs, which could reduce the stress in the implant bone area by 36.6% and optimize implant porosity, length, and diameter [18].

### 3.3. Novel Approaches in Surface Modification

No studies were found that directly applied AI to modify or analyze implant surface properties. Most research on surface treatments (e.g., roughening, coatings) remains experimental. Thus, AI's role in surface modification appears minimal to date, representing an area for future exploration.

The manual systematic search could not identify additional articles on the particular topic.

### 3.4. Predicting the Success of Osseointegration

Deep learning has begun to assist in evaluating osseointegration. One study used convolutional neural networks on postoperative radiographs to predict whether an implant had successfully osseointegrated. The AI models achieved accuracy up to approximately 89% and AUROC about 0.90% [25]. This suggests that imaging-based AI can complement traditional implant stability assessments. In general, such models “found that osseointegration of dental implants can be predicted to some extent” by analyzing radiographic images [25].

Five articles were missing from the AI conducted systematic search in the topic [26–30].

A review conducted on the topic reported that AI models could accurately assess the degree of osseointegration from radiographic images, with some models achieving high accuracy. The models rely on the subtle changes in bone-implant interfaces that are often difficult to grade by visual examination. Sensitivity ranged between 67% and 95%, and specificity between 78% and 100%, when the algorithms were trained specifically to detect signs of successful or compromised osseointegration [27].

### 3.5. Clinical Applications

#### 3.5.1. Immediate Loading

AI tools have been developed to assist in decisions about the immediate loading of implants. In an in vitro trial, Degidi and Daprile [31] trained an AI on insertion-torque curves; the AI's classification of cases as suitable for immediate loading matched experienced clinicians 99.3% of the time [31]. A later multicenter clinical study by the same group applied the AI model to 1320 implants: the AI achieved approximately 90.5% sensitivity and about 96.3% specificity for predicting immediate-loading feasibility [32]. These results indicate that AI can reliably predict primary stability from drill data, potentially guiding clinicians on when immediate loading is appropriate. All tested implants generally achieved high primary stability except in the lowest-density bone [31]. Thus, AI shows promise for enhancing immediate-loading protocols, though further large-scale validation is warranted.

No articles were missing from the AI's conducted systematic search on the topic.

#### 3.5.2. Computer-Guided Implant Placement

AI is increasingly integrated into digital planning systems. Systematic reviews report that AI algorithms improve automated segmentation of jaw anatomy (bone, sinuses, and nerves) on CBCT, facilitating precise virtual implant planning [33, 34]. For example, Macrì et al. [33] noted that AI-assisted planning demonstrated gains in precision and predictability compared to manual methods. Elgarba et al. [6] found that AI enables faster and more consistent landmark identification, although fully automated end-to-end planning is not yet realized. Case reports also highlight novel uses: one study combined AI-based CBCT segmentation with mixed-reality visualization, achieving clinically acceptable guided implant placement in simple cases [35]. In summary, AI-augmented planning and navigation enhance presurgical workflows (automating bone segmentation and guide design), but complete automation and widespread adoption remain future goals [6, 33, 36].

Four articles were missing from the AI's conducted systematic search in the topic [37–40].

#### 3.5.3. Zygomatic Implants

Advanced technology has been explored for complex zygomatic implants. A recent study tested a prototype robotic arm with dynamic navigation for placing zygomatic implants in models. The system achieved mean 3D entry/exit deviations of 1.8–2.8 mm and approximately 1.74° angular error. The authors concluded that the “errors were within the clinically acceptable limits,” although further refinement is needed before clinical use. This demonstrates that combining robotics with AI-driven navigation can feasibly increase precision in challenging implant cases. No studies specifically applied machine learning models solely to zygomatic implants beyond these guided-surgery experiments, but robotics represents a notable direction [41].

No article was missing from the AI's conducted systematic search on the topic.

#### 3.5.4. Future Directions in Implantology

Recent reviews highlight an ongoing transition toward AI-enabled implantology. Innovations include AI-driven diagnostics, predictive analytics, and robotics. For example, Karnik et al. [3] emphasize that AI and robotic systems can optimize implant positioning, foresee complications, and personalize treatment plans [3]. Although many of these technologies are still emerging, their potential is clear: AI can analyze large patient datasets to refine implant design and planning, and robots can perform or assist surgery with high precision. However, most advancements are not yet fully integrated into routine practice or commercially available [3]. Ethical and practical challenges (data quality, validation, and cost) remain topics for future research. Overall, the consensus is that AI will increasingly complement human expertise in implantology, leading toward more efficient, customized, and outcome-driven care.

No articles were missing from the AI's conducted systematic search on the topic.

#### 3.5.5. Quantitative Comparison

In total, the gold standard manual search yielded 124 articles, and 23 of them were truly relevant and could be included for the synthesis. In comparison, deep research found 114 articles, and it included 13 articles, of which two were irrelevant and nonexistent articles. Assessing the sensitivity and specificity of forming a final pool of articles for the synthesis, ChatGPT's systematic search had a high specificity (98%) and low sensitivity (47.8%). However, AI search has acceptable accuracy (88.7%), which can potentially be used for scoping research topics to test for novelty and feasibility. McNemar's test shows statistically significant differences (*p*=0.016) between manual and ChatGPT deep research function, which concludes that the current state of deep research does not allow for independent systematic search and selection.

Time spent on research topic and protocol creation, search key development, prompt preparation, and validation were similar and done parallel for both manual and deep research purposes. Effective time spent on search, selection, and validation until the final pool of articles were 19 min for deep research; 13 and 15 h for the reviewers with the manual search and selection.

## 4. Discussion

### 4.1. Summary of the Findings Regarding AI and Dental Implantology

This review finds that AI applications in implant dentistry are growing rapidly across multiple fields. In design and planning, AI algorithms have demonstrated improvements in precision (e.g., stress reduction, anatomical segmentation) [15, 33]. Our findings are in line with other literature showing high AI accuracy in implant identification and classification, suggesting that even routine diagnostic tasks may be automated [17]. The strong agreement between AI predictions and expert judgment in immediate-loading decisions is also consistent with prior modeling studies in implant stability [32]. Notably, the absence of AI-focused studies on surface modification highlights a gap; existing reviews of surface treatments emphasize material science rather than informatics. Compared to older systematic reviews (e.g., Revilla-León et al. [15]), our 2025 update confirms the “great potential” of AI models while recognizing they are still evolving. Challenges remain: AI systems require large, high-quality datasets and rigorous clinical validation before routine use. Moreover, the variability of AI models (different algorithms, input data, and study quality) complicates direct comparisons. Future research should address these limitations by standardizing protocols, integrating diverse data (imaging, biomechanics, and patient factors), and evaluating outcomes in prospective trials. In the context of the broader literature, our review supports the view that AI will supplement but not replace clinician decision-making, serving as a decision-support tool that enhances implant success rates and patient safety.

### 4.2. The Role of ChatGPT in Systematic Reviews, Deep Research, and Scientific Writing

The AI software has successfully conducted a systematic search, selected eligible articles, and done the synthesis, although it has also made numerous mistakes. First and foremost, the systematic search conducted by ChatGPT has only found 114 articles, whereas the manual search has yielded 124 articles in the same database with the same search key. The final pool of articles utilized for the synthesis by ChatGPT had also been found by the manual systematic search. The final pool contained no duplicates, and all the articles were real and could be tracked by the DOI identifier provided. The articles were in line with the research question, publication year, and adhered to the inclusion and exclusion criteria defined by the authors. However, one article did not exactly investigate AI directly; only the introduction part of the article mentioned AI in implantology, making it ineligible to be included in the synthesis [41]. The AI software initially stated that it found 13 eligible articles for the synthesis; however, the reference table only contained 11 articles. When asked of the missing articles, the software made up two nonexistent articles to account for the missing ones, which significantly lowers the trust in the capabilities of ChatGPT.

ChatGPT had already been credited as a coauthor for 18 scientific articles by the end of December 2022. In response, the chief editors of the journal “Nature” have already made a statement that no AI software meets the criteria for authorship. Therefore, they cannot be credited [42]. A group of scientists has tested whether they can spot AI-generated abstracts based on real and published research papers, but they could only identify 68% of such creations [8]. The reason it is worrying is that many AI-generated texts could find their way into the literature if reviewers are not careful enough to spot them [8]. Fake citations can also mislead peer reviewers, and compromise the chain of evidence, weaken systematic reviews, and guidelines [43]. The problem appears in medical and dental subfields as well, as an integrity risk (“reference hallucination”) noted in recent literature [44]. There are several suggestions to enhance patient safety from issues mentioned above, such as human-in-the-loop for all clinical uses (AI outputs are advisory, not authoritative) and guardrails in interfaces (uncertainty statements, blocking of unsupported citations) [45]. The editorial board of science.org has updated their policies to exclude any papers that contain texts, images, or figures generated by any LLM. However, content intentionally generated and highlighted in such papers does not fall under this rule. Using AI always needs human supervision, as it can make several mistakes, most notably, referencing a scientific study that does not exist [7].

These LLMs are evolving based on human feedback; in the past, asking the AI a clinical question may have generated a seemingly accurate answer, but someone with a deeper understanding of the topic could spot many factual or misleading errors. However, due to the numerous updates, new models, and refined questioning practices, AI can generate evidence-based answers and provide them with the necessary citations. Therefore, it is important to emphasize that due to the rapid advancements in AI and LLMs, search results and their accuracy may vary between different models; also, the specific prompts used have a significant impact on the quality of response generation. There are already software programs developed to combat AI-written summaries, essays, or reviews, by predicting the chances of generated text; however, the constant improvement of the chatbots makes it very hard to keep up with the updates. Therefore, journals should develop policies to encourage transparency in the usage of AI chatbots [46]. All in all, ChatGPT deep search is not yet ready to conduct a systematic search and synthesis; however, with the constant improvement, it might be possible in the near future (Table 2).

### 4.3. Recent Advancements in Implantology

As we can see, AI technology is not only utilized as a language model but also used in implantology [15]. From optimizing implant design and its geometric properties, predicting the chances of osseointegration based on X-rays, to segmentation and alignment of CT images to improve implant planning. The trend towards digitalization in dentistry is gaining momentum, with AI anticipated to have a significant influence on the development of the forthcoming digital systems [47]. Therefore, learning about and comprehending AI is crucial for dentists and implantologists not only to take advantage of future opportunities but also to recognize the challenges associated with its implementation.

Digital dentistry is becoming the new norm in prosthodontics and implantology; however, in implant-supported full-arch restorations, the conventional workflow is still the preferred method [48]. Recent publications in the topic have shown convincing results that the difference in precision between conventional and digital methods is dwindling constantly, which could possibly tip the scales in favor of digital workflow with time [2]. Zygomatic implants are the perfect example of digitalization in oral surgery; the difficulty lies in the positioning of the implants [49]. Digitally planned surgical guides and novel inventions like robotic assistance aims to solve such issues [3]. In the current state of language models, they are not advanced enough to be used as the main source of evidence, and to systematically assess and synthesize up-to-date literature. However, it may be useful as an additive tool to format, rephrase, or generate introductory parts based on previously prepared or newly generated text. However, deep research is a perfect tool to preliminarily screen the literature on a particular topic, to help with the decision of feasibility and novelty. It must be emphasized that any use of AI-generated content needs to be supervised and validated by experienced individuals with sufficient knowledge on the specific topic in question.

### 4.4. Strengths and Limitations

The strength of the current study is that it objectively investigates the capabilities of ChatGPT and its new plugin called deep research. The study compares the output of the software on specific topics in comparison with manual systematic search and evidence synthesis, discusses its uses and limitations, and provides possible applications. One of the limitations is that we only investigated one AI software. However, there are numerous widely available options that would provide valuable evidence on the current state of language models. The most severe limitation of such models is the generation of nonexistent references, which makes the backtracking of real references time-consuming and the output less trustworthy. Also, only one reviewer validated the results of deep research, which may distort the comparison of manual and AI searches.

### 4.5. Implications for Further Research

For future research, it is advisable to compare the output of different AI models with similar keywords and protocol to investigate the possibilities of systematic search, selection, synthesis, and risk of bias assessment. The development of AI language models is exponential, and the possibilities are limitless. Hence, their processing power may be useful in the future for methodologically rigorous studies, such as systematic reviews and meta-analyses.

## 5. Conclusions

The use of AI in scientific papers can improve the efficiency of the writing process, and ChatGPT deep research can be a great tool to preliminarily assess the feasibility and novelty of a new research question. However, there is a significant difference between the results of manual and deep research search and selection. Therefore, it is not yet suitable to reliably conduct systematic searches and to fully automate selection procedures. AI software should be used with great caution, keeping in mind its limitations, and highlighting that AI-generated content was intentionally used.

## Figures and Tables

**Table 1:** Nonexistent literature generation and reason for exclusion given by ChatGPT.

Title	Topic of investigation	Summary	Reason for exclusion
Artificial intelligence in dentistry: ethical and legal considerations for clinical practice	Ethical and legal implications of AI deployment in clinical dental practices, including implantology.	The article offers a conceptual and legal analysis of AI in dentistry, highlighting the risks of bias, accountability issues, and data privacy in AI-driven systems.	While relevant to AI in dental practice broadly, it does not provide primary data or specific findings on implant design, placement, osseointegration, or other target subtopics.
Artificial intelligence in dentistry: applications and trends	A scoping review of AI across various dental disciplines, including implantology, diagnostics, and orthodontics.	This paper offers an overview of trends in AI applications but focuses heavily on diagnostics and restorative dentistry, offering only a superficial mention of implant-related tools.	Does not provide detailed analysis, data, or evaluation of AI applications within any of the seven specified topics (implant design, surface modification, etc.).

**Table 2:** Strengths and limitations of deep research.

Strengths	Limitations
Thoroughly analyzes scientific databases	Not every article was found by deep search (~8% missing)
Accaptable understanding of the research question, inclusion, and exclusion criteria	There are still certain instances when it generates nonexistent literature
Concisely synthesizes the eligible articles and generates an appropriate discussion	Not ready yet for a trustworthy systematic search

## Data Availability

The data that support the findings of this study are available from the corresponding author upon reasonable request.
